# Editorial: Neurological, neurophysiological, psychological and psychiatric effects of high altitude and hypoxia

**DOI:** 10.3389/fphys.2023.1329084

**Published:** 2023-11-15

**Authors:** Matiram Pun, Marika Falla, Giacomo Strapazzon, Katharina Hüfner

**Affiliations:** ^1^ Department of Physiology and Pharmacology, Cumming School of Medicine, University of Calgary, Calgary, AB, Canada; ^2^ Institute of Mountain Emergency Medicine, Eurac Research, Bolzano, Italy; ^3^ Department of Neurology/Stroke Unit, Hospital of Bolzano (SABES-ASDAA), Bolzano, Italy; ^4^ Department of Anesthesia and Intensive Care Medicine, Medical University of Innsbruck, Innsbruck, Austria; ^5^ Department of Psychiatry, Psychotherapy, Psychosomatics and Medical Psychology, University Hospital for Psychiatry II, Medical University of Innsbruck, Innsbruck, Austria

**Keywords:** high altitude, hypoxia, psychiatry, psychology, neurology, neurophysiology

Mental disorders show a prevalence of 30%–40% in the general population (Steel et al., 2014), and neurological symptoms such as headache or dizziness are among the most common presenting symptoms in emergency care. Despite their high prevalence, neurological symptoms outside of headache and high-altitude cerebral edema have only been investigated to a limited extent during hypoxia and high-altitude (HA) exposure (Wilson et al., 2009). HA can also affect higher cortical functions, leading to cognitive disturbances (Falla et al., 2021). The effect of hypoxia and HA on the development of psychiatric symptoms is even less investigated (Hüfner et al., 2019). This contrasts sharply with the increasing number of individuals traveling to HA areas for leisure or occupational purposes and recent advances in the use of (intermittent) hypoxia as a treatment for various symptoms and conditions (Burtscher et al., 2023).

The aim of the current Research Topic was to collect studies on the neurological, neurophysiological, psychological, and psychiatric effects of hypoxia and HA in order to advance research in the field and bring together researchers from different specialities involved in hypoxia and HA research. Within the Research Topic, we report the results of two controlled studies performed under laboratory conditions. Such studies performed in controlled environments help greatly to clarify our understanding of physiological and pathophysiological mechanisms at HA, since HA field studies are often performed under challenging conditions and thus often involve small sample sizes or confounding factors. Importantly, reporting guidelines for HA field studies have been established in recent years (Brodmann Maeder et al., 2018) and have proven useful when conducting and reporting research from HA field studies (Hüfner et al., 2021).

Riveros-Rivera et al. report on the sex differences of daytime napping at a simulated altitude of 2,660 or 4,000 m asl in normobaric hypoxia compared to normoxia (Riveros-Rivera et al.). Fifteen healthy volunteers underwent a polysomnography, hematological, and cognitive evaluation around a 90 min midday nap. Napping in hypoxic conditions compared to normoxia lead to electrocardiogram changes (RR interval shortening) suggesting sympathetic activation in a sex-dependent way (male participants had longer RR intervals than women). The sympathetic activation was also evident with the increase of epinephrine and cortisol levels. Periodic breathing, which has been linked to a longer RR interval during sleep at high altitude (Insalaco et al., 2016), occurred mostly in the male participants during the investigated daytime nap and was present in only one female participant with polycystic ovary syndrome, suggesting a link with the testosterone level. The study suggests that napping in hypoxic conditions differentially affects sleep architecture, cardiac autonomic modulation, and respiratory pattern in male and female subjects, which could lead to more individualized recommendations during HA exposure.

Pighin et al. assess the effect of mild hypoxia on risk-taking behavior (Pighin et al.). Twenty-five healthy individuals were investigated in an adequately powered, single-blinded, and counterbalanced study design during mild normobaric hypoxia exposure (FiO_2_: 14.1%) and normoxia. The decision-making task was incorporated after 20 min of hypoxia/normoxia exposure. The participants were hypoxemic, with about ∼10% blood oxygen desaturation, and had an elevated heart rate. In the hypoxic condition, the participants were prone to taking higher risks in the betting task, although there was no effect on the gains. The study highlights that hypoxia and probably also HA exposure affect cognition and can also have an influence on the decision-making capacity of climbers with possible effects on health and safety.

Frank et al. review the role and mechanisms of hypoxia in the pathophysiology of migraine and propose how this could give new insights into the mechanisms underlying migraine and other primary and secondary headaches (Frank et al.). Specifically, the authors propose that normobaric hypoxia could be used as a “human model” for the induction of migraine and aura symptoms.

The perspective article by You et al. reviews whether and how hyperbaric oxygen preconditioning could prevent acute HA diseases. Possible mechanisms include protection of the blood–brain barrier, an inhibition of inflammatory responses, the induction of hypoxia-inducible factor, and an increase in antioxidant activity. After analyzing the existing literature, the authors acknowledge the potential of hyperbaric oxygen precondition in preventing HA diseases but also point out that the optimal protocol still needs to be established, and additional well-controlled and designed investigations are needed (You et al.).

In a separate contribution also involving the editors of this Research Topic, Hüfner et al. discuss how psychotic symptoms occurring at HA can potentially be classified in the existing diagnostic categories of the ICD-10 (World Health Organization, 2023) or Diagnostic Statistical Manual of Mental Disorders (American Psychiatric Association, 2022), such as delirium or brief psychotic disorder. The authors specifically discuss the entity of isolated HA psychosis, which consists of psychotic symptoms at HA without any other discernible psychopathological signs and symptoms (Hufner et al., 2018). In their hypothesis and theory article, the authors discuss whether specific diagnostic criteria are needed for isolated HA psychosis and offer possible suggestions.

The articles collected in this Research Topic provide new data and perspectives on the neurological, neurophysiological, psychological, and psychiatric effects of HA and hypoxia. This Research Topic offers insights into and suggestions for the design and execution of future studies to more comprehensively understand, predict, and treat the individual’s response to hypoxia and HA exposures. The effect of HA and hypoxia on individuals with pre-existing neurological or pre-existing mental disorders is still insufficiently covered in this Research Topic and in the current medical literature. Gathering evidence-based data in these populations will help to reduce the stigma associated with neurological and especially mental disorders in the mountaineering community and other populations exposed to hypoxia or HA and, subsequently, lead to an improvement in medical care and patient safety.
